# Delayed Diagnosis of Cardiac Amyloidosis Masked by Chronic Coronary Artery Disease and Ischemic Cardiomyopathy: A Case Report of an Overlooked and Underdiagnosed Etiology

**DOI:** 10.7759/cureus.63151

**Published:** 2024-06-25

**Authors:** Azeem Rathore, Vanshika Tripathi, Pankaj Mathur, Dinesh Kadariya

**Affiliations:** 1 Internal Medicine, University of Florida College of Medicine – Jacksonville, Jacksonville, USA; 2 Cardiology, University of Florida College of Medicine – Jacksonville, Jacksonville, USA

**Keywords:** cardiogenic shock, intensive care unit, echocardiography, coronary artery disease, cardiac amyloidosis

## Abstract

Cardiac amyloidosis (CA) is an infiltrative disease of the heart characterized by the deposition of insoluble protein aggregates in the myocardium. There are two subtypes of CA, and they are named after the misfolded protein. Transthyretin cardiac amyloidosis (ATTR-CA) is caused by the accumulation of the tetrameric transthyretin protein produced in the liver, and light-chain cardiac amyloidosis (AL-CA) occurs due to circulating abnormal light-chain deposition. Disease manifestation can be very non-specific, and there can be overlap with other cardiac processes. This often leads to a delay in diagnosis and a poor prognosis. Here, we present a case of delayed diagnosis of CA spanning over several years that required a multidisciplinary approach but ultimately resulted in fatality six years after the initial diagnosis.

## Introduction

Amyloidosis is a disease process that involves the accumulation of abnormal proteins known as amyloid fibrils into tissues [1]. Virtually any organ system can be affected by this process. When the myocardium of the heart is involved, the disease is known as cardiac amyloidosis. Though many kinds of proteins can be implicated in amyloidosis, transthyretin and light-chain immunoglobulins are the two major kinds of misfolded proteins that most commonly infiltrate the myocardium. Transthyretin is a tetrameric protein produced in the liver that helps carry thyroid hormone and vitamin A in the body. The two major subclasses of transthyretin amyloidosis are wild-type amyloidosis (wtATTR amyloidosis) and hereditary amyloidosis (hATTR amyloidosis) [2]. Light-chain amyloidosis is seen in plasma cell dyscrasias such as multiple myeloma, non-Hodgkin lymphoma, or Waldenstrom macroglobulinemia. Though transthyretin amyloidosis and light-chain amyloidosis are the two most common forms of cardiac amyloidosis, up to 95% of cases involve the former [1,2]. Cardiac amyloidosis manifests commonly as restrictive cardiomyopathy, with symptoms such as shortness of breath, lower extremity edema, elevated jugular venous pressure, hepatic congestion, and ascites due to right-sided ventricular failure. Other manifestations can include syncope, arrhythmia from conduction system disease, and low-output cardiac failure in advanced forms of the disease. Electrocardiograms can show a low-voltage rhythm, and echocardiography can classically have a basal-predominant hypokinesis with apical sparing (ASP) of longitudinal strain [2,3]. As these symptoms are non-specific, there is often a misdiagnosis or diagnostic delay due to other common disease processes, such as heart failure or coronary artery disease, having a similar presentation. Though treatment for cardiac amyloidosis exists, the delay in diagnosis often proves to be fatal [3].

## Case presentation

An 86-year-old male with a past medical history of chronic heart failure with reduced ejection fraction, coronary artery disease status post-multiple percutaneous interventions with stenting, moderate mitral valve regurgitation, atrial tachycardia requiring multiple radiofrequency ablations, hypertension, hyperlipidemia, and mild neuropathy underwent a routine transthoracic echocardiogram (TTE) in November of 2016. The results showed a left-ventricular ejection fraction of 30%-35%, concentric left-ventricular hypertrophy (LVH), and a “speckled appearance” of the posterior and proximal left ventricle base concerning an infiltrative process (though no ASP of longitudinal strain was obtained) (Videos 1, 2).

**Video 1:** The PLAX image reveals moderate concentric LVH with an LVEF of 30%-35%. PLAX: parasternal long axis; LVH: left-ventricular hypertrophy; LVEF: left ventricular ejection fraction

**Video 2:** The A4C view shows a speckled, bright posterior wall and proximal LV myocardium, with apical sparing. The mitral valve is mildly thickened with mild-to-moderate regurgitation. A4C: apical four-chamber; LV: left ventricle

**Figure 1:**
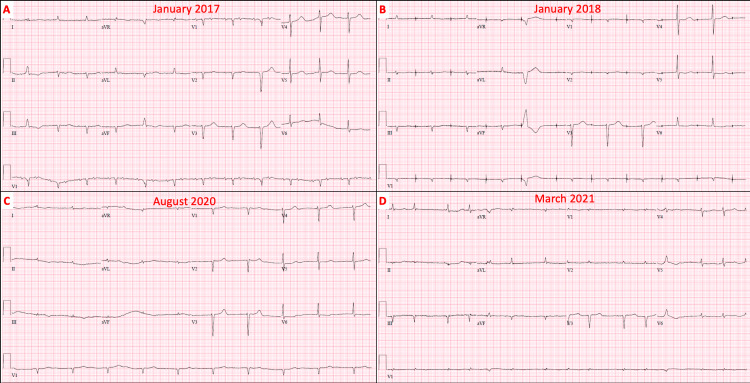
Various ECGs obtained during outpatient visits reveal the following: (A) atrial tachycardia with 2:1 conduction and low-voltage QRS; (B) atrial paced rhythm with low voltage; (C) junctional rhythm with low-voltage QRS; and (D) atrial fibrillation with low voltage. ECG: electrocardiogram

No further follow-up imaging or lab tests were performed at the time. In February 2017, the patient was admitted to the hospital for a heart failure exacerbation. A TTE was performed during the hospitalization, and the findings were stable and similar to the one completed in November 2016. However, there was no mention of an infiltrative process. The patient underwent a left heart catheterization (LHC) in July 2019, which revealed mild coronary artery disease with patent right coronary artery (RCA), left main-left circumflex (LM-LCx) stents, along with a stable pre-procedure TTE. In April 2020, the patient was admitted for a malfunctioning pacemaker. The device interrogation revealed atrial tachycardia, and a TTE performed at that time revealed similar findings. Between 2016 and 2020, the patient’s worsening cardiac function was initially attributed to be secondary to either ischemia or tachycardia-induced cardiomyopathy. In August 2020, the patient had another TTE performed, and his left-ventricular ejection fraction was noted to be decreased to 15%-20%. The patient was seen by interventional cardiology and electrophysiology, and the possibility of cardiac amyloidosis was considered given the patient’s history of multiple arrhythmias and electrocardiograms with lowering voltages (Figure 1).

**Figure 2:**
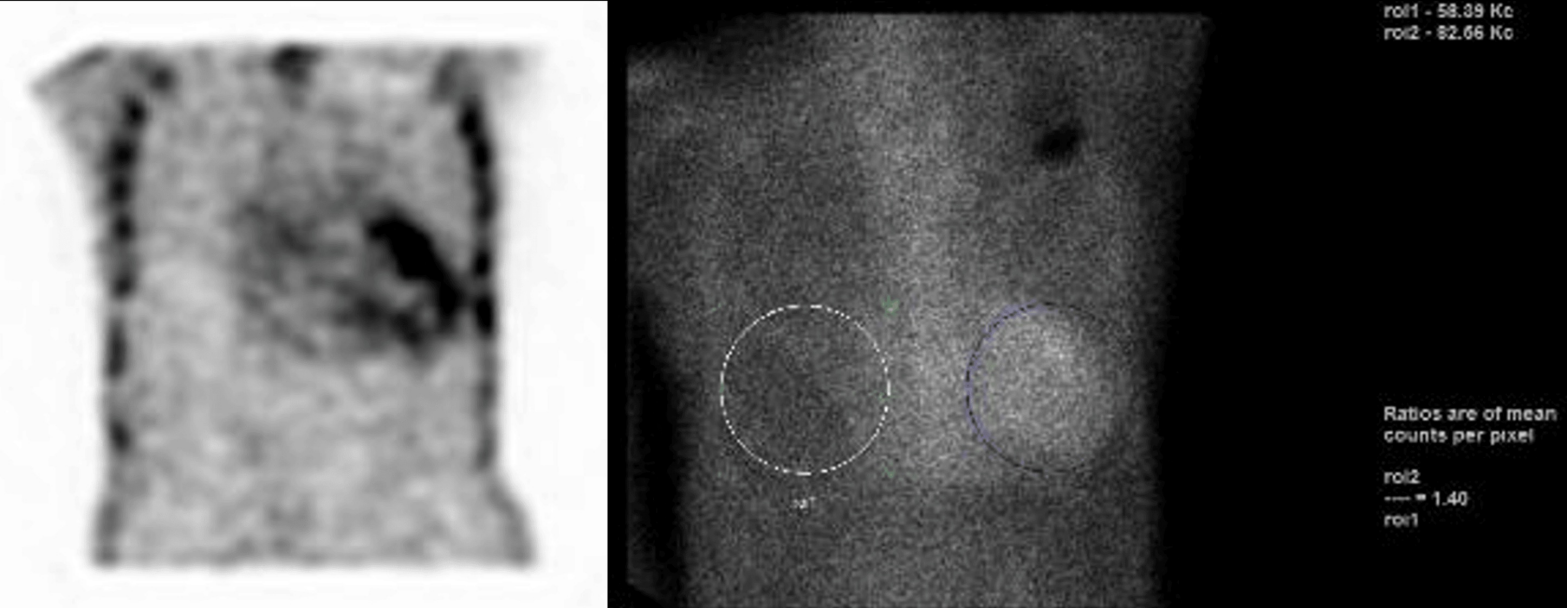
Cardiac PYP scan revealing myocardial uptake of the radiotracer that is higher than the rib uptake, indicating grade 3 myocardial uptake (left) with an H/CL ratio uptake of 1.4 (right) that collectively confirms cardiac amyloidosis. PYP: pyrophosphate; H/CL: heart-to-contralateral

A technetium pyrophosphate scintigraphy (PYP scan) was ordered to further evaluate the potential presence of cardiac amyloidosis. In October 2020, the patient was admitted for syncope, and differential diagnoses include medication-induced sequela of worsening heart failure, valvopathy, or cardiac amyloidosis. Around the same time, the patient underwent the PYP scan, which confirmed the diagnosis of cardiac amyloidosis (Figure 2).

In November 2020, the patient was evaluated by hematology-oncology, and a fat-pad biopsy with Congo-red staining confirmed transthyretin cardiac amyloidosis. Neurology assessed the patient in January 2021 and identified ongoing peripheral neuropathy raising concern for systemic amyloidosis. At this time, it was also revealed that the patient’s daughter had a long-standing history of idiopathic cardiomyopathy prompting genetic testing. The diagnosis of length-dependent sensorimotor neuropathy was subsequently made, and genetic testing confirmed heterozygous TTR mutation (*V122I* gene mutation). The patient was admitted for atrial tachycardia in March 2021 and was loaded with dofetilide for worsening episodes of tachycardia. The patient was admitted to the medical intensive care unit in March 2022 for mixed cardiogenic and septic shock. Hospice care was pursued given the patient’s poor prognosis and he eventually passed away.

## Discussion

There is often a delay in the diagnosis of cardiac amyloidosis because the disease manifestation can mimic other, more common cardiac conditions. It usually presents with right-sided heart failure symptoms such as shortness of breath, lower extremity edema, prominent jugular veins, and hepatic congestion. There must be a high degree of suspicion for an accurate diagnosis, and a multidisciplinary approach is often required. Chronic coronary artery disease can also mask and delay the diagnosis of this disease, as was the case for this patient who had multi-vessel disease [4]. Cardiology specialists initially attributed this patient’s cardiomyopathy to either worsening ischemia, poorly controlled congestive heart failure, or tachycardia-induced cardiomyopathy. Diagnostic clues suggestive of cardiac amyloidosis include unexplained dyspnea, LVH with low-voltage electrocardiograms, and basal predominant hypokinesis with apical sparing seen on echocardiography. Additionally, speckle-tracking echocardiography can be helpful in diagnosing cardiac amyloidosis, as it detects the typical pattern of basal predominant hypokinesis and ASP, providing further non-invasive imaging of myocardial tissue abnormalities [5].

According to a study, the presence of interventricular septal and left-ventricular posterior-wall thickening is of moderate sensitivity (57%) but good specificity (90%). However, in that same study, neither the presence of hypertension nor the absence of low voltage on the electrocardiogram eliminated a diagnosis of cardiac amyloid deposition [6]. A low-voltage ECG coupled with LVH is reasonably specific for amyloid; however, it is not highly sensitive and is even less so in TTR than in AL cardiac deposition [6,7]. This reflects on the decision made initially in this case not to pursue an infiltrative cause of the disease and encourages medical professionals to reduce the threshold for investigating amyloidosis in patients with similar symptom profiles. Furthermore, recent studies indicate that cardiac amyloidosis is more common than previously thought, as evidenced by several autopsy studies [8,9]. These studies highlight the importance of considering cardiac amyloidosis in patients with unexplained heart failure and suggest that the prevalence of this condition has been historically underestimated.

Considering the grim prognosis, a correct and timely diagnosis is essential and potentially life-saving. Early diagnosis of affected persons using easily accessible noninvasive diagnostics is crucial, as transthyretin amyloid cardiomyopathy (ATTR-CM) treatment may be most effective when started before substantial cardiac dysfunction occurs [10]. Diagnostic tests such as bone scintigraphy, cardiac magnetic resonance imaging (CMR), and serum or urine electrophoresis with immunofixation should be considered when suspecting cardiac amyloidosis [10,11]. Moreover, the PYP scan should be interpreted in the context of negative blood tests for AL amyloidosis. A negative blood test for AL and a positive PYP scan are sufficient for a non-invasive diagnosis of TTR amyloidosis [12,13].

Additionally, endomyocardial biopsy can confirm the diagnosis when non-invasive methods are inconclusive [13]. However, in this case, a fat-pad biopsy was pursued for confirmation at the recommendation of hematology, likely to definitively rule out other potential causes and confirm the diagnosis beyond any doubt. This decision, however, was not made via a multidisciplinary approach and, in retrospect, likely was unnecessary. Another aspect that requires reflection relates to the lack of CMR being performed despite the fact that it has become a standard of care for the evaluation of cardiomyopathy, as it can help identify amyloid deposits and assess the extent of cardiac involvement [12]. Indeed, CMR likely could have led to an earlier diagnosis and workup of cardiac amyloidosis in this case.

If ATTR-CM is found, genetic sequencing of the TTR gene is essential to distinguish between normal and wild-type diseases for two reasons. Firstly, it can be used for prognostication, while the confirmation of the *V122I* gene should trigger genetic counseling and potential screening of family members [11]. Secondly, it can guide treatment options because newer disease-modifying therapies are specifically targeted for certain gene types due to the varying underlying pathophysiology of disease types [11-15]. The recently approved drugs, such as tafamidis, patisiran, and inotersen, have shown promise in stabilizing the TTR protein and halting disease progression [14,15].

Evidence of amyloid deposition in tissues is required to diagnose cardiac amyloidosis, but bone scintigraphy and CMR can bridge this diagnostic gap [12,13]. Bone scintigraphy, particularly with Tc-99m PYP, can be highly sensitive and specific for ATTR amyloidosis, making it a valuable tool in early detection [12,13]. Early detection and appropriate therapy can help halt the progression of cardiac damage, but delayed diagnosis is frequent and may require multiple visits to different subspecialty physicians, including interventional cardiologists, electrophysiologists, neurologists, and hematology oncologists for an accurate diagnosis to be made [1,14,15]. Even after diagnosis, the prognosis can be poor, as in the case of our patient, who died six years after initial concern for cardiac amyloidosis.

## Conclusions

Amyloidosis is a severe disorder where misfolded proteins form insoluble amyloid fibrils in organs such as the heart, nervous system, soft tissues, kidneys, and gastrointestinal tract. Cardiac amyloidosis is defined as the deposition of insoluble misfolded protein in the myocardium of the heart, resulting in dysfunction. The two subtypes include transthyretin cardiac amyloidosis caused by misfolded transthyretin protein deposition and light-chain amyloidosis caused by abnormal circulating immunoglobulin light-chain deposition. Due to non-specific symptoms, overlap with other heart conditions, and lack of awareness, cardiac amyloidosis is often delayed in diagnosis and carries a poor prognosis.
